# Management of pulmonary tuberculosis patients in an urban setting in Zambia: a patient's perspective

**DOI:** 10.1186/1471-2458-10-756

**Published:** 2010-12-07

**Authors:** Chanda Mulenga, David Mwakazanga, Kim Vereecken, Shepherd Khondowe, Nathan Kapata, Isdore Chola Shamputa, Herman Meulemans, Leen Rigouts

**Affiliations:** 1 Tropical Diseases Research Centre, Biomedical Sciences Department, P. O. Box 71769, Ndola, Zambia; 2 Institute of Tropical Medicine, Department of Microbiology, Mycobacteriology Unit, 2000, Antwerp, Belgium; 3 Institute of Tropical Medicine, Department of Parasitology, Helminthology Unit, 2000, Antwerp, Belgium; 4 Ministry of Health, National Tuberculosis and Leprosy Program, Lusaka, Zambia; 5 Tuberculosis Research Section, Laboratory of Clinical Infectious Diseases, National Institute of Allergy and Infectious Diseases, National Institutes of Health, Bethesda, MD 20892, USA; 6 University of Antwerp, Department of Sociology and Research Centre for Longitudinal and Life Course Studies (CELLO), 2000, Antwerp, Belgium; 7 University of Antwerp, Faculty of Biomedical, Pharmaceutical and Veterinary Sciences, Department of Biomedical Sciences, 2000, Antwerp, Belgium

## Abstract

**Background:**

Zambia continues to grapple with a high tuberculosis (TB) burden despite a long running Directly Observed Treatment Short course programme. Understanding issues that affect patient adherence to treatment programme is an important component in implementation of a successful TB control programme. We set out to investigate pulmonary TB patient's attitudes to seek health care, assess the care received from government health care centres based on TB patients' reports, and to seek associations with patient adherence to TB treatment programme.

**Methods:**

This was a cross-sectional study of 105 respondents who had been registered as pulmonary TB patients (new and retreatment cases) in Ndola District between January 2006 and July 2007. We administered a structured questionnaire, bearing questions to obtain individual data on socio-demographics, health seeking behaviour, knowledge on TB, reported adherence to TB treatment, and health centre care received during treatment to consenting respondents.

**Results:**

We identified that respondents delayed to seek treatment (68%) even when knowledge of TB symptoms was high (78%) or when they suspected that they had TB (73%). Respondent adherence to taking medication was high (77%) but low adherence to submitting follow-up sputum (47%) was observed in this group. Similarly, caregivers educate their patients more often on the treatment of the disease (98%) and drug taking (100%), than on submitting sputum during treatment (53%) and its importance (54%). Respondent adherence to treatment was significantly associated with respondent's knowledge about the disease and its treatment (p < 0.0001), and with caregiver's adherence to treatment guidelines (p = 0.0027).

**Conclusions:**

There is a need to emphasise the importance of submitting follow-up sputum during patient education and counselling in order to enhance patient adherence and ultimately treatment outcome.

## Background

Tuberculosis (TB) continues to be a major health problem in Zambia, despite a long running National Tuberculosis and Leprosy Programme (NTLP). In 2007, the World Health Organization (WHO) estimated the TB burden in Zambia to be at 60,337 cases (all forms of TB) [1]. The TB control efforts have been hampered by the high level of human immunodeficiency virus (HIV) infection, especially in urban settings where prevalence is estimated to be 19.7% [2]. As a result the number of TB and HIV cases threatens to overwhelm the capacity of the general health systems. HIV-TB co-infection rates in Zambia have been estimated at 70% [1].

Zambia adopted the WHO recommended Directly Observed Treatment Short course (DOTS) strategy as its primary approach in TB control in 1993 and has officially reported 100% DOTS coverage in all nine provinces since 2003 [3]. A good functioning primary health care system is crucial in the implementation of DOTS. In Zambia, the NTLP activities have been integrated into the primary health care services. The decentralisation of TB treatment services has provided for more responsibility at the lower levels of the health care system and in the face of an overwhelming TB case-load, this move has proved to be beneficial to the practical implementation of the programme. Despite the human resource challenges, the use of treatment supporters and community volunteers in the implementation of DOTS has contributed to the improvement in cure rates over the past decade from 67% in 2000 to the global target of 85% by 2006 [3]. The goal of the Zambian NTLP is to prevent and control TB through the provision of quality diagnostic and treatment services for TB and TB/HIV- infected individuals at all levels of the health care delivery system [4].

Assessing access to quality of healthcare service delivery is complex and multidimensional and will depend on several aspects that are both patient/community-related and/or health systems/service related. Several questions could be considered in this vein, for example, are patients seeking help when they are sick, and when they do seek healthcare, are they getting the appropriate care they require when they need it and ultimately, is this care effective when they get it? Understanding the factors that affect or influence care actions in different settings, will ultimately result in an improvement in healthcare delivery.

Although there are several reports about health seeking behaviour of TB patients and factors related to their delay in seeking health care, compliance to treatment and the role of these factors in treatment outcome, only a few studies describe patient experience in accessing TB care throughout treatment. This study describes and assesses the care received by pulmonary TB patients from government health care centres, and the association with patient adherence to TB treatment based on previous TB patients' reports. The study also alludes to patient's attitude to seek health care for TB.

## Methods

### Study design and population

This was a cross-sectional study of subjects who had been treated for pulmonary TB through the NTLP at government health centres in Ndola, an urbanized city on the Copperbelt Province of Zambia with an estimated population of 374,757 persons [5], representative of many urban towns along the line of rail in Zambia. At the time of the study, the Ndola District Health Management Team (NDHMT) provided health care services through 26 health centres. All the health centres provided TB treatment and care (treatment centres), but only six were able to perform Acid Fast Bacilli (AFB) smear microscopy (diagnostic centres).

### Sampling and sample size

The sampling frame comprised the names of all the smear-positive TB patients, new and retreatment cases, registered in the TB microscopy laboratory registers at the six diagnostic centres between January 2006 and July 2007, as a record of all smear-positive patients undergoing treatment in the 26 treatment centres in that period. Those that had received treatment from private clinics or hospitals and children less than 18 years of age were not included. A sample of 105 respondents was randomly selected from the sampling frame. The sample size was calculated using Epi Info 3.5.1 (Centers for Disease Control and Prevention, Atlanta, GA, USA). Based on pre-test results, we expected a frequency of patient compliance and adherence to treatment of 50%±10%, at a confidence interval of 95%, and non-response level of 10%, and therefore estimated a sample size of 105 as sufficient.

### Data collection, management and analysis

Initial contact with the selected respondents was made through the TB focal persons at the health centres. Trained research assistants from the Tropical Diseases Research Centre (TDRC), interviewed consenting participants using a structured questionnaire at their homes. The questionnaire, bore questions to capture individual data on socio-demographics, knowledge on TB, health seeking behaviour, adherence to TB treatment, and reported health centre care during treatment. Most of the questions were closed ended. The questionnaire was pre-tested before use and modifications incorporated in the final version.

The collected data was entered in an MS Access database using Epi Info™3.5.1 (Centers for Disease Control and Prevention, Atlanta, GA, USA), with in-built consistency and range checks. The database was converted to SAS^® ^9.2 (SAS Institute Inc., Cary, NC, USA) for recoding where necessary and final analyses. Fisher's exact Chi-squared test was used to examine associations of factors. A p ≤ 0.05 was considered significant.

### National Guidelines for management of TB

The management of TB patients in Zambia has been standardised under guidelines provided by the NTLP [6]. Except for the seriously ill and identified multidrug resistant (MDR) cases, TB patients are treated on an ambulatory basis. Patients are instructed to pick up medication at TB treatment centres once or twice a week during the intensive phase and once monthly during the continuation phase. The national guidelines stipulate that treatment during the intensive phase should be under direct observation by a trained treatment supporter - usually a relative, while the continuation phase can be self-administered but with monthly supervision from the health centre. Patient education is an important aspect of TB treatment management and is also included in the guidelines to improve cure rates and compliance. Further, as part of patient monitoring and follow up, microscopy is to be repeated at 2, 5 and 8 months. To ensure and improve compliance to sputum follow-up, it is the duty of treatment centres to (1) ensure patients make follow-up visits and submit sputum specimens as required (2) deliver sputum specimens to the nearest diagnostic centre for microscopy and (3) collect microscopy results from diagnostic centres and make available to patients for appropriate care. Patients do not visit diagnostic centres themselves.

### Conceptual framework

The following concepts were used to make analysis.

#### Respondent treatment adherence

Respondents that reported to have completed eight months of taking medication without interruption, and submitted sputum at least twice post diagnosis - one time point being at eight months - were considered to have adhered to the treatment programme.

#### Care giver treatment guidelines adherence

Caregivers that were reported by the respondents to have enquired about patient's TB history, provided patient information (on TB disease and its treatment, how to take medication, the requirement to submit follow-up sputum during treatment and the importance of submitting follow-up sputum), and gave the patient an opportunity to ask questions, were considered to have adhered to the TB treatment guidelines.

#### Respondent knowledge

Respondents that were able to name the correct mode of TB transmission, at least two correct symptoms of TB and knew the importance of treatment completion and sputum submission were considered to be knowledgeable about the disease and its treatment.

#### Health centre systems access

Health centre delivery systems were considered to be adequate if respondents reported that: the distance to the health centre was less than 30 minutes walk from their home, he/she was commenced on TB treatment not more than 5 days post laboratory diagnosis, and he/she used the same clinic for follow-up treatment and follow-up sputum submission.

### Ethical consideration

Approval for the study protocol was obtained from the Ethics Committee at TDRC. Approval and support were also obtained from the Director of the NDHMT. Consenting respondents were asked to sign an informed consent following an explanation of the study. Interviewers were not part of the health care system. Respondents were assured of anonymity and confidentiality.

## Results

### Respondent characteristics and health seeking attitudes

Basic Respondent socio-demographic characteristics are shown in Table 1. Other results showed that 68% of respondents waited for one month or more since the onset of symptoms before going to the health centre. When asked why they waited that long, most of the respondents (76%) thought the symptoms will go away. The most common response for how they coped with symptoms prior to visiting the health centre was self-treatment (64%). Most of the respondents (98%) only presented at the health centre when they were feeling very sick. When asked if they suspected that they had TB, 30 respondents (29%) responded in the affirmative. However, 73% of these respondents still waited for at least one month before going to the health centre.

**Table 1:** Socio-demographic characteristics of the respondents (N = 105)

		n	%
Sex			
	Female	50	48
	Male	55	52
			
Age (years)			
	15 - 24	13	12
	25 - 34	33	31
	35 - 44	29	28
	45 - 54	16	15
	55 - 64	7	7
	> 65	7	7
			
Marital Status			
	Married/Cohabiting	58	55
	Single	23	22
	Divorced/Separated	11	11
	Widowed	13	12
			
Education			
	None	8	8
	Primary	44	42
	Secondary	50	48
	Tertiary	3	3
			
Employment			
	Formal	18	17
	Informal	44	42
	Housewife	13	12
	Dependent	15	14
	Unemployed	15	14
			
Distance to clinic			
	5-10 minutes	41	39
	20-30 minutes	43	41
	45 minutes	9	9
	1 hour	10	10
	Too far to walk, need to get bus	2	2
			
Previous episode of TB			
	Yes	23	22
	No	82	78

#### Respondent treatment adherence

When respondents were asked if they had stopped taking their medication at some point during treatment, 22% said yes, and the most common reason for stopping was that the respondent felt better (55%). Among respondents that were asked the number of times they submitted sputum after initiation of treatment, 32% reported submitting sputum at three time points, 25% at two time points, whilst 43% submitted sputum only once post treatment initiation. Two thirds (67%) of the respondents reported submitting sputum at the end of treatment, (eight months). Adherence to treatment of respondents is shown in Table 2 (A).

**Table 2:** Distribution of respondent and caregiver adherence and health systems access in Ndola, Zambia (N = 105)

	n	%
**A. Respondent adherence to treatment programme**		
Respondents that complied and adhered to treatment programme	45	43
1. Respondents that completed medication without stopping at any point	81	77
2. Respondents that submitted sputum as required	50	48
		
**B. Caregiver adherence to treatment guideline**		
Respondents whose caregivers adhered to treatment guidelines	26	25
1. Respondents whose caregivers enquired about their TB history	88	84
2. Respondents whose caregivers educated them on:		
the disease and its treatment	103	98
how to take medication	105	100
requirement of submitting follow-up sputum	56	53
the importance of follow-up sputum submission	57	54
3. Respondents who were given an opportunity to ask questions	49	47
		
**C. Respondents' knowledge on the disease**		
Respondents that demonstrated knowledge on the disease and its treatment	30	29
1. Respondents that gave the correct mode of TB transmission	73	70
2. Respondents that gave at least two correct symptoms of TB	82	78
3. Respondents that knew the importance of treatment completion	94	90
4. Respondents that knew the importance of follow-up sputum submission	57	54
		
**D. Health centre systems access**		
Respondents that reported adequate healthcare systems access	84	80
1. Respondents who reported the distance to the health centre as being too far	84	80
2. Respondents who reported commencing treatment within a week of diagnosis	105	100
3. Respondents who reported using the same clinic for treatment and sputum submission	77	73

#### Care giver treatment guidelines adherence

The majority of respondents (84%) confirmed that they were asked if they had suffered from TB previously before commencement of TB treatment. To the questions enquiring whether the health-worker explained how to take the medication and whether the instructions were clear, nearly all responded favourably. When asked if the health worker informed them at the initiation of treatment that they would have to submit more sputum samples during treatment, 53% said yes; all of whom reported that the health centre staff explained to them the importance of submitting follow-up sputum specimens. Forty-nine (47%) respondents reported that they were given an opportunity to ask questions for clarifications. Table 2 (B) shows performance of caregivers' adherence to treatment guidelines.

#### Respondent knowledge and awareness of TB

When asked to name some symptoms of TB, a significant proportion of the respondents (78%) was able to mention at least two symptoms, with cough being the most identified symptom (89%). A considerable number (69%) of the respondents correctly knew the mode of transmission of TB, however, 13% incorrectly cited using the same utensils. Knowledge of the importance of completing medication for eight months was high (89%) but knowledge for the importance of submitting follow-up sputum was lower (55%). Ninety-one percent of the respondents reported that they knew they were cured of their last TB episode, but when asked how they knew they were cured, reasons ranged from feeling better (80%), the fact that they took medication for eight months (15%), to that laboratory results were negative (4%). Table 2 (C) shows performance of respondents with regards to knowledge and awareness of TB. Most respondents (71%) did not suspect that they had TB despite the large number (85%) naming cough as one of the symptoms they experienced.

#### Healthcare systems access

TB treatment centres appeared relatively close to the respondents' homes: 80% lived within 30 minutes walk, 18% lived within an hour's walk and 2% said it was too far to walk and needed to take a bus. Respondents were also asked how long after being diagnosed with TB it took before starting medication; all the respondents reported that they were started on treatment within one week of diagnosis, with 86% starting within two days.

When respondents were asked if they had used the same clinic for their follow-up visits and drug collection throughout treatment, affirmative responses were 87%. Respondents were further asked if they had submitted their follow-up sputum samples to the same clinic they went for reviews and collected drugs from, and 73% said yes. Table 2 (D) shows performance of health centres with regards to access as reported by the respondents.

#### Factors significantly associated with respondent adherence

The results showed that, using our conceptual framework, respondents' adherence to treatment was not only significantly associated with respondent's knowledge about the disease and its treatment (p < 0.0001), but also with reported caregivers' adherence to treatment guidelines (p = 0.0027) and reported adequate healthcare systems access (p < 0.0001) (Table 3).

**Table 3:** Respondent adherence associations to Caregiver adherence, Respondent knowledge and Health system accessibility (N = 105).

	Respondent adherence to treatment programme		
	
Characteristics	Adhered	Did not adhere	*P value
**A. Caregiver adherence to treatment guidelines**			
Did not adhere to guidelines	27	52	
Adhered to guidelines	18	8	0.0027
			
**B. Respondents' knowledge on TB**			
Not knowledgeable	20	55	
Knowledgeable	25	5	< 0.0001
			
**C. Health centre systems access**			
Not good/not efficient	0	21	
Good/efficient	45	39	< 0.0001

*P values are based on Fisher's exact chi square test.

Further analyses showed that caregivers explaining the importance and schedule of follow-up sputum submission was significantly associated with respondents' adherence to sputum submission as required (p < 0.0001), but not with respondents' completing medication for eight months (p = 0.0562).

## Discussion

The success of a national TB program is multi-faceted and complex. Community awareness; patients' adherence to treatment; patient access to quality of care through competent healthcare staff who are able to provide quality of care through prompt diagnosis and referral, prescription of correct treatment regimens and treatment follow-up; and accessible TB services, are important components of a successful TB program.

Consequently, it is important that people in communities are aware and able to suspect TB in persons who show signs and symptoms suggestive of the disease, such as prolonged cough, persistent fevers, and weight loss. Maybe not surprising, as previous TB patients our respondents showed a good level of knowledge on the symptoms and modes of transmission of TB, attributable to caregiver education during treatment. However, our study revealed vast differences in knowledge regarding the importance of treatment completion compared to knowledge of the importance of follow-up sputum submission; whereas, nearly 90% knew the importance of treatment completion, only 57% knew the importance of the latter, reflective of the low importance given to the relevance of education on this issue. Similarly, other studies have shown that most TB patients know the importance of treatment completion [7-9]. According to our conceptual framework, overall knowledge of the disease was low, mainly due to the low knowledge gap in the role of sputum microscopy in TB treatment by the respondents.

Despite the high knowledge levels of TB symptoms shown in our study, most respondents not only, reported not to have suspected they had TB, but also reported that they delayed seeking care (even when they suspected they had TB). Whereas it is possible that respondents were truly unaware of TB symptoms prior to TB treatment, several other studies have shown that there are various reasons why patients delay seeking care at a health centres. Loss of income, health centre systems or staff attitudes, stigma of the HIV association, severity of disease, lifestyle, for example, alcohol abuse, are among the many explanations [9-13]. The most common reasons in our study, 'I was thinking the symptoms will go away' or 'I did not think it was serious' also appear to be common in different settings [8,12]. This may be reflective of the commonly practiced self-treatment, which may ameliorate initial symptoms thus temporarily masking the severity of disease and consequently 'buy them time' to continue with their daily income generating endeavours. Only 17% of our study population were in formal employment suggesting that for most respondents an income was dependent on their daily efforts and therefore may not afford the time at the health centre. Further, the period of the study, were the early days of scaling up of free antiretroviral therapy in Zambia and so people may still have been feeling helpless against HIV infection.

Our results showed that only 47% of respondents reported to have submitted follow-up sputum at least twice post diagnosis and that 67% reported submitting follow-up sputum at the end of treatment. These results may be cause for concern because sputum re-examination at the end of the patient's treatment is a much stronger indicator of treatment success than 'treatment completion. Further, data in one of our studies in this population, has shown that among subjects who experienced another episode of TB within one year of completing treatment, there were more who harboured the same *M. tuberculosis *strain as that of the previous episode (relapses/treatment failures) than those that had a different strain (re-infection) (unpublished data). Furthermore, our study showed a high proportion of respondents taking of drugs for the complete period of treatment (89%) with a notable proportion (22%) reporting stopping medication at some point during treatment. Over half (55%) cited that they stopped because they were feeling better, similar to many other studies [14,8,15].

The role of the health worker on patient compliance has been described many times [16-18]. Patient counselling and good communication [19,20] can improve patient compliance. Our study showed high levels of patient satisfaction when it came to health provider explanation regarding medication. However, we did not see the same positive response with regards to health provider explanation on the role of follow-up sputum submission. Only about half of the respondents reported that they were informed about the requirement (53%) and importance (54%) of submitting follow-up sputum. In fact, these two parameters were shown to be significantly associated with respondent adherence (p < 0.0001 for both). A study in Egypt demonstrated that adherence to recommended sputum smear microscopy schedule was significantly associated with treatment success [21]. Our study also showed that respondent adherence to treatment was significantly associated with respondent's knowledge about the disease and its treatment (p < 0.0001) in contrast to other studies [22,8].

Moreover, caregivers' communication skills fell short on account of dialogue, giving the patient a chance to ask questions, an important aspect in patient management that ensures patient understanding of disease and treatment. The effects of non-dialogue counselling were demonstrated in a study in Madagascar where reported lack of opportunity to ask questions by patient was significantly associated with non-adherence [16].

Other features of the health system, like distance, convenience of TB services (microscopy, antiretroviral treatment services), how long it takes to see the clinician, prompt diagnosis and referral of TB patients presenting with TB-related symptoms at primary health care facilities, may have an effect on patient access to healthcare. Distance to the health centre for this population was not an issue. Delays in the commencement of treatment have been documented in some settings [23], our study, however, showed that all the respondents were given medication within one week of diagnosis, with 84% commencing treatment within two days post laboratory diagnosis. The NTLP in Zambia has given full responsibility of sputum transportation plus obtaining and communicating results for each patient, to the treatment centres. This not only reduces on the number of patients, who remain undiagnosed following initial health centre visit, but also removes the inconvenience and added travel costs from the patients. The majority of our respondents reported that they used the same treatment centre for sputum submission. Our results indicate that facility-service related factors may not be the main issue in patients' access to TB care in Ndola, unlike the study from KwaZulu-Natal where systems failure was reported as contributing to the ineffectiveness of the National Tuberculosis Program [24].

Admittedly, because this study asked questions about past events, participants' recall may have biased our results. In addition, since the interview was anonymous to ensure complete confidentiality, we were not able to go back to the patient's data files to verify the self-reported data. Nevertheless, the implied cure rate for this sample population is comparable to the average cure rate data for the same period from Ndola. Another limitation for this study is that we did not establish from the respondents how long it took for laboratory results to be available for diagnosis, a factor that could well contribute to delay in TB patient care. However, enquiries from TB focal persons indicated a turnaround time for lab results ranging from the same day to a week. Further, our study did not include all components of TB treatment and care in the National Guidelines and consequently, other components that contribute to this package have not been discussed. Lastly, it is well known that respondents usually consider the interviewer to represent authority or the healthcare system and therefore tend to bias their answers in the way they expect they should to please the interviewer. Consequently, although the study made efforts to use researchers from outside the respondents' healthcare system, it is difficult to completely remove this perception in communities.

## Conclusions

In conclusion, TB treatment systems appear to be well in place in NDHMT. However, taken together, these results suggest that closer monitoring systems on guidelines adherence at health centres may need strengthening and more patient counselling on treatment of disease and importance of sputum submission may improve cure rates.

## Competing interests

The authors declare that they have no competing interests.

## Authors' contributions

CM was involved in the design and implementation of the study, and drafted the manuscript.

ICS conceived and designed the study and critically revised the manuscript. HM, DK and KV performed statistical analysis and critically revised the manuscript. SK and NK critically revised original study design and the manuscript. LR supervised the implementation and critically revised the manuscript. All the authors have read and approved the final manuscript.

## Pre-publication history

The pre-publication history for this paper can be accessed here:

http://www.biomedcentral.com/1471-2458/10/756/prepub
